# SUBJECTIVE AGE, DEPRESSIVE SYMPTOMS, AND COGNITIVE FUNCTION: DOES CHRONOLOGICAL AGE MATTER?

**DOI:** 10.1093/geroni/igae098.2658

**Published:** 2024-12-31

**Authors:** Juhyeong Lee, Giyeon Kim

**Affiliations:** University of British Columbia, Seoul, Kyongsang-bukto, Republic of Korea; Chung-Ang University, Seoul, Republic of Korea

## Abstract

This study examined the mediating effect of depressive symptoms between subjective age and cognitive function among Korean older adults, with a focus on the moderating effects of chronological age. Using data from the 2020 National Survey of Older Koreans, a nationally representative sample of individuals aged 65 or older (N=9,836) was examined. Subjective age was calculated by subtracting perceived old age from chronological age, with higher values indicating an older subjective age. Depressive symptoms were measured using the Short Form of Geriatric Depression Scale (SGDS-K), and cognitive function was assessed using the Korean version of Mini-Mental State Examination for Dementia Screening (MMSE-DS). A moderated mediation analysis was conducted by PROCESS (Model=59) for SPSS. Results showed that depressive symptoms significantly mediated the relationship between subjective age and cognitive function among Korean older adults. Particularly, an older subjective age was associated with lower depressive symptoms (B=-.0420, p<.001). Depressive symptoms were related to poor cognitive function (B=-.2372, p<.001). The direct effect of subjective age on cognitive function was also significant (B=-.1017, p<.001). Furthermore, chronological age moderated the indirect effects, demonstrating that subjective age was more strongly associated with lower depressive symptoms at younger chronological ages, whereas both subjective age and depressive symptoms were more strongly linked to poorer cognitive function at older chronological ages. The findings underscore the importance of considering both chronological and subjective age in understanding the interplay between depressive symptoms and cognitive function among Korean older adults.

